# Antioxidant Properties, Bioactive Compounds Contents, and Chemical Characterization of Two Wild Edible Mushroom Species from Morocco: *Paralepista flaccida* (Sowerby) Vizzini and *Lepista nuda* (Bull.) Cooke

**DOI:** 10.3390/molecules28031123

**Published:** 2023-01-23

**Authors:** El Hadi Erbiai, Abdelfettah Maouni, Luís Pinto da Silva, Rabah Saidi, Mounir Legssyer, Zouhaire Lamrani, Joaquim C. G. Esteves da Silva

**Affiliations:** 1 Biology, Environment, and Sustainable Development Laboratory, Higher School of Teachers (ENS), Abdelmalek Essaadi University, Tetouan 93000, Morocco; 2 Chemistry Research Unit (CIQUP), Institute of Molecular Sciences (IMS), Department of Geosciences, Environment and Territorial Planning, Faculty of Sciences, University of Porto, Rua do Campo Alegre s/n, 4169-007 Porto, Portugal

**Keywords:** *Paralepista flaccida*, *Lepista nuda*, bioactive compounds, biomolecules, antioxidant activity, Moroccan mushroom, wild edible mushroom

## Abstract

Mushrooms have been consumed for centuries and have recently gained more popularity as an important source of nutritional and pharmaceutical compounds. As part of the valorization of mushroom species in northern Morocco, the current study aimed to investigate the chemical compositions and antioxidant properties of two wild edible mushrooms, *Paralepista flaccida* and *Lepista nuda*. Herein, the bioactive compounds were determined using spectrophotometer methods, and results showed that the value of total phenolic content (TPC) was found to be higher in *P. flaccida* (32.86 ± 0.52 mg) than in *L. nuda* (25.52 ± 0.56 mg of gallic acid equivalents (GAEs)/mg of dry methanolic extract (dme)). On the other hand, the value of total flavonoid content (TFC) was greater in *L. nuda* than in *P. flaccida*, with values of 19.02 ± 0.80 and 10.34 ± 0.60 mg of (+)-catechin equivalents (CEs)/g dme, respectively. Moreover, the ascorbic acid, tannin, and carotenoids content was moderate, with a non-significant difference between the two samples. High-performance liquid chromatography–mass spectrometry (HPLC-MS) analysis allowed the identification and quantification of thirteen individual phenolic compounds in both *P. flaccida* and *L. nuda*, whereas *p*-Hydroxybenzoic acid was recognized as the major compound detected, with values of 138.50 ± 1.58 and 587.90 ± 4.89 µg/g of dry weight (dw), respectively. The gas chromatography–mass spectrometry (GC-MS) analysis of methanolic extracts of *P. flaccida* and *L. nuda* revealed the presence of sixty-one and sixty-six biomolecules, respectively. These biomolecules can mainly be divided into four main groups, namely sugars, amino acids, fatty acids, and organic acids. Moreover, glycerol (12.42%) and mannitol (10.39%) were observed to be the main chemical compositions of *P. flaccida*, while *L. nuda* was predominated by linolelaidic acid (21.13%) and leucine (9.05%). *L. nuda* showed a strong antioxidant property, evaluated by DPPH (half maximal effective concentration (EC_50_) 1.18–0.98 mg/mL); β-carotene bleaching (EC_50_ 0.22–0.39 mg/mL); and reducing power methods (EC_50_ 0.63–0.48 mg/mL), respectively. These findings suggested that both mushrooms are potential sources of various biomolecules, many of which possess important biological activities which are interesting for the foods and pharmaceuticals industry.

## 1. Introduction

Mushrooms have been consumed for centuries due to their nutritional and medicinal benefits. In terms of nutritional value, the fruiting bodies of mushrooms are known to be rich in high-quality protein, essential and non-essential amino acids, have a high proportion of unsaturated fatty acids, a good source of fiber, and a higher amount of carbohydrates, and are also full of micronutrients, such as vitamin B complex, and a high level of mineral elements that are essential for human health [1,2]. In medicinal terms, several studies have demonstrated that mushrooms contain a wide variety of bioactive compounds, such as alkaloids, carotenoids, enzymes, fats, glycosides, organic acids, phenolics, polysaccharides, proteins, terpenoids, tocopherols, vitamins, and volatile compounds in general. These compounds from mushrooms have shown a wide range of biological activities, including antioxidant, antibacterial, antifungal, antitumor, immunomodulating, cardiovascular-protective, antiviral, antiparasitic, antifibrotic, anti-inflammatory, antidiabetic, anti-atherosclerotic, hypoallergenic, antiatherogenic, hypoglycemic, hepatoprotective, and hypotensive properties [1,2]. Consequently, mushrooms have become more attractive as functional foods and as a source of nutraceuticals and pharmaceutical compounds.

Oxidative stress is involved in many diseases, as a trigger or associated with complications. Most of these diseases appear with age, which leads to serious pathologies such as cardiovascular and neurodegenerative diseases, cancer, diabetes, metabolic syndrome, and digestive disease [3]. Many important molecules with antioxidant properties can help the endogenous defense system against oxidative stress caused by the excess of reactive oxygen and nitrogen species (ROS and RNS) [4]. Wild mushrooms contain different antioxidants such as phenolic compounds, tocopherols, ascorbic acid, carotenoids and more other molecules which could be extracted to be used as functional ingredients, namely against chronic diseases related to oxidative stress [4,5].

*Paralepista flaccida* (Sowerby) Vizzini, (2012) is a wild edible basidiomycete mushroom belonging to the order *Agaricales* and the family *Tricholomataceae* [6]. It is known to form fairy rings [7]. *Paralepista* species were generally assigned either to the genus *Lepista* or *Clitocybe*, until 2012 when Alfredo Vizzini and Enrico Ercole published a paper that confirmed by molecular analysis that these mushrooms are a separate clade from other *Lepista* species (such as *Lepista nuda*) and also from *Clitocybe* species (such as *Clitocybe fragrans*) [8]. This genus is recognized by Species Fungorum [9], and the Global Biodiversity Information Facility [10].

The naming of this mushroom is complicated, and some references generally listed the *flaccida* and *inversa* forms as separate species, in which the case of *inversa* is distinguished because it grows under conifers rather than broad-leaved trees, has a shinier cap surface, and is more rigid (less flaccid), which is according to our collected samples. However, other mycologists are considering *inversa* as a variety of *flaccida*, and, finally, some modern authors merge the two into one [7,9,10,11,12,13,14].

*P. flaccida* is saprophytic species growing naturally on humus-rich soil and compost under deciduous trees, while the *inversa* form grows under conifer needles. It is frequently distributed in Europe [12] and has also been reported wildly in forests of *Quercus*, *Cedrus*, *Acacia*, and *Pinus* in diverse areas of Morocco including Chefchaouen, Ktama, Tangier, Lalla Mimouna, Middle Atlas and Rabat [15,16,17,18,19,20].

*Lepista nuda* (Bull.) Cooke, (1871) (also called *Clitocybe nuda*, commonly known as blewits) is an edible basidiomycete mushroom belonging to the same order and family as *P. flaccida*. It is a saprotrophic species found in both deciduous and mixed forest areas in Europe, North America, Asia and Australia [21,22]. Due to its special fragrance and delicate texture, *L. nuda* has been cultivated in several countries, including France, Holland, Britain, and Taiwan [21]. In Morocco, *L. nuda* has been found widely under *Quercus*, *Cedrus*, and *Pinus* trees in several sites, including Chefchaouen, Dardara, Oued Laou, Bouhachem, Gourougou, Ain Sferjla, Oued Cherrat, Lalla Mimouna, Mamora, Rabat and also in coastal plateau from Essaouira to Tangier [16,18,19,20,23,24,25].

Many studies on the chemical compositions and biological activities of mushrooms have been made in northern Mediterranean countries concerning the species growing in this region. However, as far as we know, there are few studies on mushrooms in southern countries, especially in Morocco, which is considered one of the richest Mediterranean countries in terms of biodiversity [25,26,27]. 

Several studies have been carried out on the chemical compositions and biological activities of *L. nuda* [2,22], while few data were reported about *P. flaccida* which were in the case of *inversa* form [2,28]. However, as we know no studies were reported on these two species growing in Morocco, except one study which was performed on the total phenolic and antioxidant activity of *L. nuda* collected from Natural Parc of Bouhachem [25].

The objective of the present study was to investigate the chemical compositions and antioxidant activity of two wild edible mushrooms, *Paralepista flaccida* and *Lepista nuda*, collected from northern Morocco. Herein, the contents of the bioactive compounds including total phenolic, total flavonoid, total ascorbic acid, total tannin, and total carotenoids contents (β-carotene and lycopene) were determined using a UV-Visible spectrophotometer, while high-performance liquid chromatography-mass spectrometry (HPLC-MS) was used for the identification and quantification of phenolic compounds, and gas chromatography-mass spectrometry (GC-MS) for biomolecules identification. Moreover, the antioxidant properties were evaluated by three different assays, including DPPH radical-scavenging, β-carotene bleaching inhibition, and reducing power assay.

## 2. Results and Discussion

### 2.1. Extraction Yield 

As presented in Table 1, the extraction yields of methanolic extracts of *P. flaccida* (30.32 %) and *L. nuda* (31.69 %) were statistically similar to each other’s, while lower than the previous yield in the Portuguese *Lepista inversa* (39%) which was reported by Heleno et al. [29].

### 2.2. Estimation of Bioactive Compounds

The bioactive compound contents in the studied mushroom samples were estimated using a UV-Visible spectrophotometer, and the results are presented in Table 1.

Total phenolic contents in the methanolic extract were observed to be significantly important in both tested species, although *P. flaccida* was shown to have a higher amount, with the value of 32.86 mg GAE/g of dme, which is higher than the previous studies by Heleno et al., (3.60 mg) [29] and Vaz et al., (10.8 mg in ethanolic extract) [30] in *Lepista inversa*. Similarly, *L. nuda* content (25.52 mg) was noted to be higher than several works from Morocco, Turkey, Portugal and Turkey, with values of 11.83, 7.7, 6.31, and 4.18 mg GAE/g of dme, respectively [25,31,32,33], which was close to the amount in the given results in the Argentinian (27.34 mg) [34] and the Indian mushrooms (23.77 mg) [35].

Concerning total flavonoid contents, the methanolic extract of *L. nuda* was given, statically, as a more important content than *P. flaccida*, with the values of 19.02 and 10.34 mg CE/g of dme, respectively. However, Barros et al. [32] (3.36 mg CE/g dme) and Sharma et al. [35] (2.47 mg quercetin equivalent/g dme) found lower flavonoid contents in *L. nuda* than in the present study.

Regarding ascorbic acid content, the fruiting body of samples presented a moderate result and there were no significant differences between *P. flaccida* (1.27 mg/g) and *L. nuda* (1.31 mg/g). The amount of ascorbic acid in *L. nuda* was observed to be higher than the values of 0.34 and 0.23 mg/g obtained in the previous studies [32,35], respectively. However, ascorbic acid was not detected in the work by Lkay Koca et al. [31]. 

The amount of tannin content in *P. flaccida* was found to be significantly similar to *L. nuda*, with the values of 2.67 and 2.26 mg CE/g of dw, respectively. To our knowledge, there were no previous studies on the tannin content of both samples. 

As shown in Table 1, β-carotene and lycopene contents were observed to be present statistically in small quantities in comparison with the premier bioactive compounds. However, the values of β-carotene and lycopene from *L. nuda* were higher than the previous study from India [35] (0.39 and 0.20 µg/100 g), while smaller than the one reported in the Portuguese sample [32] (2.52 and 0.98 µg/g).

Overall, the content of bioactive compounds determined in the studied wild edible mushrooms from Morocco was very important, although these compounds have been previously estimated in many other mushrooms and are known for their strong antioxidant capacity [5].

### 2.3. Phenolic Compounds by HPLC–MS Analysis

**Figure 1:**
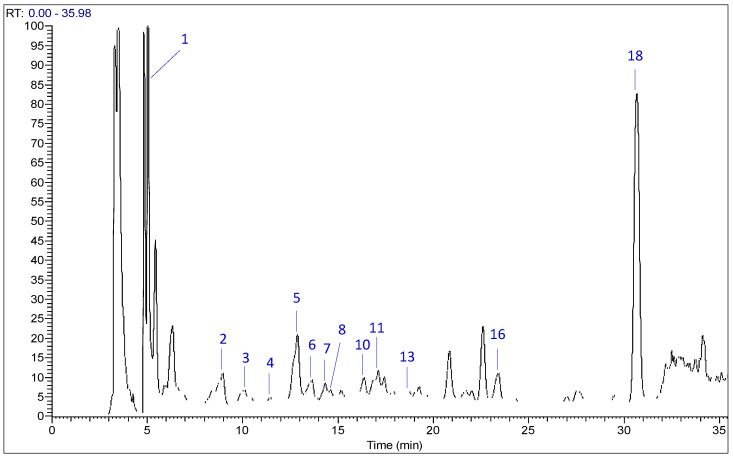
HPLC–MS chromatogram of phenolic compounds in *Paralepista flaccida* extract detected at 280 nm.

The identification and quantification of individual phenolic compounds in fruiting body extracts of *P. flaccida* and *L. nuda* were performed using the HPLC–MS technique. The chromatogram illustrating the phenolic compounds peaks in *P. flaccida* and *L. nuda* is shown in Figure 1 and Figure S1, respectively, whereas Table 2 gives the amounts of the thirteen compounds identified and quantified by using standards and their mass spectra. The HPLC-MS results showed that *p*-hydroxybenzoic acid was recognized as the major phenolic compound identified and quantified in both mushrooms *P. flaccida* and *L. nuda*, with values of 138.50 and 587.90 µg/g dw, respectively. Chlorogenic acid (136.30 µg/g) was classified as the second main compound in *P. flaccida*, followed by gallic acid (132 µg/g) and cinnamic acid (124.20 µg/g), while catechin (400.20 µg/g), ellagic acid (362.60 µg/g) and chlorogenic acid (327.60 µg/g) were listed as the second, the third and the fourth main phenolic compounds detected in *L. nuda* extract, respectively. The lowest component that had been detected was syringic acid for both *P. flaccida* and *L. nuda*, with values of 11.25 and 8.57 µg/g dw, respectively. However, the phenolic compounds rutin, vanillin, rosmarinic acid, salicylic acid and quercetin were not detected in either sample. Statistically, and except gallic acid, all phenolic compounds characterized in the current work showed a significant difference in the comparison between the two tested mushrooms (Table 2).

There have been a few investigations on phenolic compounds of the studied mushrooms, whereas *P. flaccida* phenolics characterization was only reported by the study of Vaz et al. [36] under the name *Lepista inversa*, without detecting any compounds in their samples; however, *L. nuda* individual phenolic compounds were analyzed in three previous research works from two countries (Portugal and Argentina). Herein, from Portugal, Pinto et al. [37] identified two compounds, *p*-hydroxybenzoic (sample from wild pine forest: 100 µg/g dw and from the wild oak forest: 150 µg/g) and cinnamic acids (from wild pine forest: trace and from the wild oak forest: 10 µg/g), with their concentrations significantly lower than the present work, while the study by Barros at al. [38] detected three phenolic acids which are protocatechuic: *p*-hydroxybenzoic and *p*-coumaric acids, with the values of 33.57, 29.31 and 3.75 µg/g dw, respectively. Regarding Argentina, Toledo et al. [34] did not find any of the phenolic compounds analyzed (gallic, *p*-hydroxybenzoic and *p*-coumaric acids) in their *L. nuda*. The phenolic compounds from mushrooms have already been studied in several species, and it was reported that these compounds have been attributed to different biological activities such as antioxidant, antimicrobial and antitumor activities [5,39].

### 2.4. Biomolecules by GC–MS Analysis

**Figure 2:**
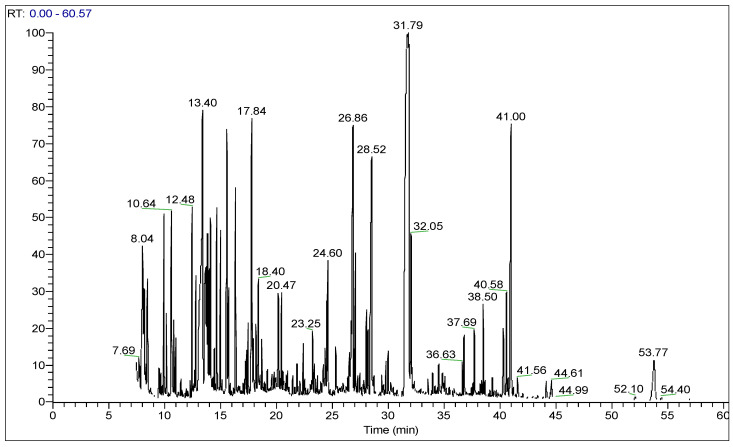
GC–MS chromatogram of *L. nuda* derivatized methanolic extract.

The chemical compositions of the fruiting bodies’ methanolic extracts after their derivatization were established by GC–MS, a powerful tool for qualitative and quantitative analysis of various compounds present in natural products and the technique widely used in medical, biological, and food research [40]; the summarized results of this analysis are represented in Tables S1–S5. The GC–MS chromatogram of *P. flaccida* (Figure S2) and *L. nuda* (Figure 2) revealed the presence of sixty-one and sixty-six biologically active compounds, respectively. The identified biomolecules can be mainly divided into five main groups of constituents of each sample, namely sugars, amino acids, fatty acids, organic acids, and the five composed of rest groups, whereas sugars (52.51%) and fatty acids (29.72%) were observed to be the main chemical group in *P. flaccida* and *L. nuda*, respectively (Table 3). Glycerol (12.42%), mannitol (10.39%) and linoleic acid (9.67%) were recognized as major chemical compositions of *P. flaccida*, while *L. nuda* was predominated by linolelaidic acid (21.13%), leucine (9.05%) and mannitol (5.05%). The two main compounds detected in this study, mannitol and linoleic acid, were previously considered antioxidants [41,42]. Alongside nutritional values, the biomolecules identified in both mushrooms could be responsible for various pharmacological actions such as antioxidant, anti-inflammatory, antimicrobial, antiviral and antitumor activities. 

As presented in Table S1, the contents of sugar compositions of methanolic extracts of the two analyzed mushrooms were strong and diverse. *P. flaccida* extract contained 21 compounds which were dominated by glycerol (12.42%), mannitol (10.39%) and trehalose (8.58%). Likewise, *L. nuda* methanolic extract was composed of 16 components, in which mannitol (5.16%), threitol (4.16%) and trehalose (4.13%) were the most abundant sugar compounds detected. Heleno et al. previously reported the presence of two sugar compounds, trehalose and mannitol, in *L. inversa* [43]. The presence of the two main sugars in *L. nuda*, mannitol and trehalose, were also observed in several previous studies [31,38,41]. Moreover, glucose, rhamnose, mannose, and xylose were the four monosaccharides quantified in *L. nuda* from India without detecting galactose and fructose [35]. Trehalose, a naturally occurring nontoxic disaccharide, functions as an antioxidant and may be useful to treat many chronic diseases, involving oxidative stress [44].

Concerning fatty acids, *L. nuda* methanolic extract contained the major diversity (11) of compounds, representing 29.72% of the total of compounds identified, whereas linoelaidic (21.13%), palmitic (4.49%) and stearic acids (1.78%) were the major fatty acids detected (Table S2). In contrast, only four fatty acids were detected in *P. flaccida,* which were predominated by linoleic (9.67%) and palmitic (1.63%) (Table S2). Several previous works studied the fatty acids of *L. nuda*, and all of them reported that linoleic acid was the main compound identified, which was not detected in our mushroom [34,35,37,45]. According to these previous studies on *L. nuda* and our *P. flaccida* result, two studies from Bulgaria and Portugal also found linoleic acid as the main fatty acid determined in the species’ *inversa* form (*Lepista inversa*) [28,43]. 

Regarding amino acids, the major diversity of amino acids was observed in *P. flaccida* (15 amino acids), while there was less in *L. nuda*, with eight compounds (Table S3). Gamma-aminobutyric acid (3.04%), glutamine (1.99%) and threonine (1.42%) were classified as the main amino acids detected in *P. flaccida*. For *L. nuda*, leucine, threonine and alanine represented the majority of the amino acids identified, with percentages of 9.05%, 2.69% and 2.15%, respectively. To our knowledge, there have been no previous studies on the amino acids of *P. flaccida* or of *inversa* form; however, one study was performed on *L. nuda* from India with the identification of four amino acids, namely aspartic acid, arginine, tyrosine and proline [35].

For organic acids, GC–MS analysis of the derivatized methanolic extracts showed the presence of eight compounds in *P. flaccida* and eleven compounds in *L. nuda* (Table S4). The *P. flaccida* was predominated by 3,4-dihydroxybutanoic (2.59%), malic (2.26%), succinic (1.85%) and citric (1.83%) acids, while acetoacetic (2%), oxalic (1.66%), maleic (1.44%) and lactic (1.32%) acids were observed to be the highest presented organic acids in *L. nuda*. Three analyses were realized on organic acids in *L. nuda*, and the results demonstrated that quinic and oxalic acids were listed as the main compounds in the three studies [34,37,46]. Contrary to our work, quinic and fumaric acids were not detected in *L. nuda*; citric acid was not detected in a study from Portugal [46], and citric and malic acids were not identified in the previous work from Argentina [34]. Organic acids may have a protective role against various diseases due to their antioxidant activity (such as in the case of tartaric, malic, citric or succinic acids), being able to chelate metals or to delocalize the electronic charge coming from free radicals [46].

Alongside sugars, fatty acids, amino acids and organic acids, the GC–MS analyses of derivatized methanolic extracts of the studied mushrooms showed that the samples also contained many other biologically active compounds belonging to the group of alcohols, steroids, nucleic acids, lipids, glycerides, etc. (Table S5). Ergosterol was noted to represent 1.97% and 1.61% of total biomolecules in *P. flaccida* and *L. nuda*, respectively. This biomolecule is the most abundant sterol found in mushrooms, and it has several biological activities including antioxidant, anti-inflammatory, anti-hyperlipidemic, anti-tyrosinase and antimicrobial activities [47,48].

### 2.5. Antioxidant Activity

Natural antioxidants have become scientifically interesting compounds due to their many benefits for human health [49]. There are numerous methods available to determine the antioxidant capacity of extracts or pure compounds. Herein, the antioxidant activity of methanolic extracts of the two Moroccan mushrooms *P. flaccida* and *L. nuda* were evaluated spectrophotometrically using three different assays: DPPH radical scavenging, β-carotene/linoleate, and Ferricyanide/Prussian blue activity. The antioxidant results are expressed in EC_50_ values, as summarized in Table 4. In addition, the results have been graphically represented in Figures S3–S5. The methanolic extracts of *P. flaccida* and *L. nuda* showed a strong antioxidant capacity, which was in agreement with the important amount of phenolic compounds and other bioactive compounds found in both mushrooms. These important results were significantly different with Trolox, a standard that was used as a control. On the other hand, the strongest antioxidant capacity was observed in *P. flaccida* extract using a β-carotene bleaching inhibition assay with the value of 0.22 mg/mL (lower EC_50_ value), and in the same mushroom the lowest antioxidant activity by using DPPH radical-scavenging activity with the value of 1.18 mg/mL (higher EC_50_ value) was noted.

Concerning DPPH radical-scavenging activity (Figure S3), the results showed that the two studied samples exhibited significant free radical reducing capacity. Herein, the methanolic extract of *L. nuda* gave higher antioxidant capacity than *P. flaccida* extract, with EC_50_ values of 0.98 and 1.18 mg/mL, respectively. A previous study was released on *L. nuda* from Morocco, and the antioxidant activity was 10.60 mg of Trolox equivalent per gram of lyophilized mushroom. Moreover, several works from other countries, namely Portugal, Argentina, Turkey and India, evaluated the DPPH radical-scavenging activity of *L. nuda* extracts and the EC_50_ values ranged between 2.16 and 16.20 mg/mL, which were significantly higher than our values [31,32,34,35,37]. Another work from Portugal, by Heleno et al., noted that *L. inversa* gave the EC_50_ value of 10.57 mg/mL, which was highly different from our results [29]. This important radical-scavenging activity is due to the high content of total phenolic and flavonoids found in the studied mushrooms [5].

Regarding the β-carotene-linoleate bleaching assay (Figure S4), the biomolecules existing in the methanolic extract of the two mushrooms were able to inhibit the discoloration of β-carotene and have demonstrated strong antioxidant properties. The methanolic extract of *P. flaccida* revealed significantly higher antioxidant activity than the *L. nuda* extract, with values of 0.22 and 0.39 mg/mL, respectively, which were more effective than *L. inversa* (1.80 mg/mL) from Portugal reported previously by Heleno et al. [29]. Furthermore, recent studies have also demonstrated the antioxidant activity of *L. nuda* extract using β-carotene-linoleate bleaching assay and the results were observed to be lower than our samples, with higher EC_50_ values which were between 3.53 and 14.24 mg/mL [32,34,37]. These important β-carotene-linoleate bleaching results could be due to the high quantity of carotenoids and other major biomolecules found in the methanolic extract of *P. flaccida* and *L. nuda*.

For reducing power by Ferricyanide/Prussian blue assay (Figure S5), the natural antioxidant compounds exiting in methanolic extracts of the two edible Moroccan mushrooms were able to convert Fe^3+^ into Fe^2+^ and, therefore, exhibited high reducing power with EC50 values of 0.48 mg/mL for the *L. nuda* and 0.63 mg/mL for *P. flaccida*. Our extracts have given a strong reducing power in comparison with previous results by Heleno et al. in *L. inversa* (2.9 mg/mL) [29], and with the ones reported in various works on *L. nuda* extracts, in which their EC_50_ values ranged between 0.75 and 4.21 mg/mL [32,34,35,37]. This finding of reducing power could be related to the ability of biomolecules found in the samples to reduce Fe^3+^ [50].

Overall, the investigated edible mushrooms are sources of powerful antioxidants such as phenolic compounds, ascorbic acid, carotenoids, and other bioactive compounds, which could be used against diseases related to oxidative stress, dermatological applications, cosmetics, and as supplements in the food industry [29]. 

## 3. Materials and Methods

### 3.1. Standards and Reagents

N,O-Bis(trimethylsilyl)trifluoroacetamide (BSTFA), alkane standards (C_8_-C_20_ and C_21_-C_40_), meta-Phosphoric acid, 2,6-Dichloroindophenol sodium salt hydrate, l-ascorbic acid, (+)-catechin, vanillin reagent, Folin–Ciocalteu’s phenol reagent, (±)-6-Hydroxy-2,5,7,8-tetramethylchromane-2-carboxylic acid (Trolox), β-carotene, Tween 40, linoleic acid, iron (III) chloride, sodium hydroxide, sodium nitrite, and phenolic standards including, caffeic acid, catechin, chlorogenic acid, cinnamic acid, ellagic acid, ferulic acid, gallic acid, methylparaben, *p*-coumaric acid, *p*-hydroxybenzoic acid, protocatechuic acid, quercetin, rosmarinic acid, rutin, salicylic acid, syringic acid, vanillic acid, and vanillin were purchased from SIGMA-ALDRICH, Co., (St. Louis, MO, USA). Acetonitrile, ethyl acetate, hydrochloric acid fuming 37%, pyridine, aluminum chlorure, and sodium chloride were obtained from Merck KGaA (Darmstadt, Germany), and 2,2-diphenyl-l-picrylhydrazyl (DPPH) was from Alfa Aesar (Ward Hill, MA, USA). Acetone, n-hexane, and hexane were purchased from CABLO ERBA Reagent, S.A.S (Val de Reuil Cedex, France). Methanol and all other chemicals and solvents were of the highest commercial grade and obtained from Honeywell (St. Muskegon, MI, USA).

### 3.2. Mushroom Material

The edible mushrooms *P. flaccida* and *L. nuda* were harvested from Koudiat Taifour forest, a Biological and Ecological Interest Site (SIBE) (35°40′45.4″N 5°17'36.3"W 180 m of altitude) in northwestern Morocco during January 2018, under *Quercus suber*, *Pinus halepensis*, *Eucalyptus rostrata* and *Pistacia lentiscus* trees. The identifications of the harvested species were undertaken in the Biology, Environment, and Sustainable Development (BEDD) laboratory at the École Normale Supérieure (ENS) of Tetouan, Morocco, and were based on macroscopic and microscopic characterizations and ecological conditions. These identifications were made according to the two determination keys [51,52]. Voucher specimens were deposited at the herbarium of the BEDD laboratory, Department of Matter and Life Sciences, ENS of Tetouan, Morocco. The fruiting bodies were immediately cleaned, weighed, cut into small pieces, air-dried, and reduced to a fine powder (20 mesh).

### 3.3. Preparation of Crude Methanolic Extracts

The methanol extraction was carried out following the previous work by Barros et al. [32], with some modifications. A total of 1 g of fine-dried mushroom power (20 mesh) was extracted by stirring with 20 mL of methanol at 25 °C at 150 rpm for 24 h and filtered through Whatman N °4 paper. The residue from the filtration was extracted again, twice, using the procedure described earlier. The combined methanolic extracts were evaporated at 40 °C to dryness. Then, the dried extracts were weighed and stored at −81 °C for further use. The extraction yield was calculated for each studied species. This preparation and all the further works were carried out at the Faculty of Sciences of the University of Porto, Portugal.

### 3.4. Estimation of Bioactive Compounds

The contents of bioactive compounds, including total phenolic compound content (TPC), total flavonoid content (TFC), total ascorbic acid content (TAAC), total tannin content (TTC), and total carotenoids contents (β-carotene (Tβ-CC) and lycopene (TLC)), in fruiting bodies of *P. flaccida* and *L. nuda* were determined by spectrophotometry using the same conditions, equipment and procedures described previously by Erbiai et al. [26]. 

TPC was determined by Folin–Ciocalteu assay. Briefly, one ml of extract methanolic solution was mixed with 5 mL of Folin–Ciocalteu reagent and 4 mL of sodium carbonate solution (7.5%). The tubes were vortex mixed for 15 s and allowed to stand for 30 min at 40 °C in the dark. Then, the absorbance of the solution was measured at 765 nm against the blank. The results were expressed as milligrams of gallic acid equivalents (GAE) per gram of dry methanolic extract (dme).

TFC was determined by using an aluminum chloride colorimetric method, based on the formation of a complex between aluminum chloride and the C-4 keto group and either the C-3 or C-5 hydroxyl group of flavones and flavonols. The intensity of the pink color was measured at 510 nm using a UV-Visible spectrophotometer against the blank, which contained all reagents except extract samples. The results were expressed as mg of (+)-catechin equivalents (CEs) per gram of dme.

TAAC was determined using a method based on the reaction of ascorbic acid existing in the extract with the reagent 2,6 dichlorophenolindophenol. Meta-phosphoric acid (1%) was used for ascorbic acid extraction. The absorbance was measured at 515 nm against a blank. The results were expressed as mg of ʟ-ascorbic acid equivalents (AAEs) per gram of dw.

TTC of the sample powder was assayed by the Vanillin-HCL method, which is a method specific to dihydroxyphenols and particularly sensitive to molecules containing meta-substituted, di- and tri-hydroxybenzene. The absorbance of color developed was measured at 500 nm against the blank. The TTC was expressed as mg of (+)-catechin equivalents per gram (CEs/g) of dme.

Tβ-CC and TLC were determined following a method based on the mixture of methanol extract and acetone-hexane (4:6). The solution absorbance (A) was measured at 453, 505, 645, and 663 nm using a UV-Vis spectrophotometer. Tβ-CC and TLC were calculated according to the following equations: *Lycopene* (mg/100 mL*)* = [(0.0458 A_663_) + (0.372 A_505_) − (0.0806 A_453_)]; *β-Carotene* (mg/100 mL) = [(0.216 A_663_) − (0.304 A_505_) _+_ (0.452 A_453_)].

### 3.5. Phenolic Compounds Analysis by HPLC–MS

The extraction and analysis of individual phenolic compounds of *P. flaccida* and *L. nuda* were carried out following the same procedure, conditions and HPLC equipment used in our previous published work [26]. Briefly, the phenolic extract was analyzed by high-performance liquid chromatography-mass spectrometry (HPLC–MS). Chromatographic separation was accomplished using Acclaim™ 120 reverse phase C18 columns (3 µm 150 × 4.6 mm) thermostatted at 35 °C, and peaks were detected at 280 nm as the preferred wavelength. The mobile phase used was composed of 1% acetic acid and 100% acetonitrile. The identification of phenolic compounds in the samples was characterized according to their UV-Vis spectra and identified by their mass spectra and retention times in comparison with commercial standards. Quantification was made from the areas of the peaks recorded at 280 nm by comparison with calibration curves obtained from the standard of each compound. The results were expressed in µg per gram of dry weight (dw).

### 3.6. Biomolecules Analysis by GC–MS

Before GC–MS analysis, the crude methanolic extracts of each mushroom (10 mg) were derivatized by adding 100 µL of anhydrous pyridine and 100 µL BSTFA, and the mixture was heated at 80 °C for 25 min, then the mixture was diluted with 200 µL chloroform [53,54]. The derivatized solution was analyzed by using Gas Chromatography (GC) (Trace 1300 gas chromatography; Thermo Fisher Scientific, Waltham, MA, USA) linked to a mass spectrometry (MS) system (ISQ single quadrupole mass spectrometer; Thermo Fisher Scientific) and automatic injector. The GC separation was conducted with a TG5-MS capillary column (60 m × 0.25 mm i.d.; 0.25 µm film thickness) with a non-polar stationary phase (5% Phenyl 95% dimethylpolysiloxane). The injection and detector temperature were made at 300 °C using splitless injection mode (1:10). Helium was used as a carrier gas at a flow rate of 1.2 mL/min. The oven temperature was programmed from 40 °C (2 min) to 200 °C at a rate of 6 °C/ min (2 min), and then at a rate of 6 °C/min (6 min) up to 300 °C. The total run time was 65 min. MS conditions were: electron ionization mass spectra were set at 70 eV, the mass ranged from 50 to 650 amu, and the ion source temperature was 300 °C. Retention indices were calculated for all components, using a homologous series of known standards of alkanes mixture (C_8_–C_20_ and C_21_–C_40_) injected in conditions equal to sample ones. Identification of components of mushroom extracts was based on retention indices (RI) relative to alkanes, with those of authentic compounds and with the spectral data obtained from the databases of the National Institute Standard and Technology (NIST) and PubChem Libraries of the corresponding compounds. Data acquisition was operated by Software Thermo XcaliburTM 2.2 SP1.48, and data analysis was performed using NIST MS Search 2.2 Library 2014.

### 3.7. Evaluation of Antioxidant Activity

The antioxidant activity of methanolic extracts from the two edible mushrooms *P. flaccida* and *L. nuda* was evaluated by three different assays, including DPPH radical-scavenging, reducing power, and β-carotene bleaching inhibition assay, and by following the same procedures, equipment and conditions used previously by Heleno et al. [29]. The extract concentration providing 50% of antioxidant capacity or 0.5 of absorbance (EC_50_) was calculated from the graphs of antioxidant activity percentages (DPPH, and β-carotene/linoleate assays) or absorbance at 690 nm (ferricyanide/Prussian blue assay) against extract concentrations. Trolox was used as a reference standard.

DPPH radical-scavenging activity (RSA) of the samples was determined using the stable free radical DPPH (1.1-diphenyl-2-picrylhydrazyl). The absorbance was measured at 517 nm using a UV-Vis spectrophotometer against a blank. The RSA was calculated as a percentage of DPPH discoloration using the equation: RSA (%) = [(A_DPPH_ − A_Sample_)/A_DPPH_] × 100, where A*_DPPH_* is the absorbance of the DPPH solution and A*_Sample_* is the absorbance of the test extract.

For the β-carotene-linoleate bleaching assay, the antioxidant activity of the methanolic extracts was carried out using the β-carotene linoleate model system, in which the presence of antioxidants in the extracts and their capacity to neutralize the linoleate free radicals avoids β-carotene bleaching. The absorbance was measured immediately at zero-time at 470 nm against a blank, and measured for the second time at 120 min. A control containing methanol instead of the extract was realized in parallel. β-carotene bleaching inhibition was calculated using the following formula: (%) = (β-carotene content after 2 h of the assay/initial β-carotene content) × 100.

Reducing power by Ferricyanide/Prussian blue assay, the methodology of which is based on the capacity to convert Fe^3+^ into Fe^2+^, the absorbance of the solution was measured at 690 nm using a UV-Vis spectrophotometer against a blank containing the same solution mixture without mushroom extract.

### 3.8. Statistical Analysis

Three samples were used, and all assays were carried out in triplicate. Extraction yield, bioactive compounds, and antioxidant activity values were expressed as mean ± standard deviation (SD). The statistical significance of the data was made with a one-way analysis of variance (ANOVA), followed by post hoc Tukey’s multiple comparison tests with α = 0.05 using GraphPad Prism 8.0.1 software (San Diego, CA, USA).

## 4. Conclusions

This research work constitutes the first report on the chemical characterizations and antioxidant properties of the two wild edible mushrooms *P. flaccida* and *L. nuda* from southern Mediterranean countries and, in particular, from Morocco. The fruiting bodies of the studied samples demonstrated an important content of bioactive compounds, namely phenolic compounds (individual and total contents), ascorbic acid and carotenoids. In addition, the GC–MS analysis of *P. flaccida* and *L. nuda* extracts revealed the presence of more than sixty biologically active compounds for each. On the other hand, the two edible mushrooms showed strong antioxidant properties by using three assays: DPPH radical scavenging activity, inhibition of β-carotene bleaching, and ferric-reducing power. The highly considered antioxidant capacity of the samples could be related to their richness of bioactive compounds. In general, the findings may encourage more people from southern Mediterranean countries to consume edible mushrooms as food due to their benefits on human health. They may also allow researchers to make the valorization of mushrooms from these regions an interesting objective for their investigation to open up new perspectives in nutritional and pharmaceutical research, and to contribute to discovering novel antioxidant agents and medicaments which can be used for the treatment of many diseases.

## Figures and Tables

**Table 1:** Extraction yield and bioactive compound contents in the dried fruiting body of mushroom studies ^1^.

Bioactive Compounds	*P. flaccida*	*L. nuda*	One-Way ANOVA *
Extraction yield (%)	30.32 ± 1.14	31.69 ± 2.04	0.4736
Total phenolic (mg GAE/g dme)	32.86 ± 0.52 ^a^	25.52 ± 0.56 ^b^	<0.0001
Total flavonoid (mg CE/g dme)	10.34 ± 0.06 ^b^	19.02 ± 0.80 ^a^	<0.0001
Ascorbic acid (mg AAE/g dw)	1.27 ± 0.06	1.31 ± 0.03	0.9048
Tannin (mg CE/g dw)	2.67 ± 0.04	2.26 ± 0.19	>0.9999
β-Carotene (µg/g dme)	0.30 ± 0.02	0.64 ± 0.01	0.9982
Lycopene (µg/g dme)	0.23 ± 0.01	0.38 ± 0.01	>0.9999

^1^ Values are expressed as means ± SD of three independent measurements. * *p* < 0.05 indicates that the mean value of at least one component differs from the others. For each mushroom sample, means within a line with different letters differ significantly (*p* < 0.05).

**Table 2:** Phenolic acids and related compounds characterized by HPLC–MS ^1^.

N°.	Phenolic Compounds	*P. flaccida* (µg/g dw)	*L. nuda* (µg/g dw)	One-Way ANOVA *
1	Gallic acid	132 ± 1.79 ^a^	131.7 ± 1.11 ^a^	0.9955
2	Protocatechuic acid	79.91 ± 2.02 ^b^	97.28 ± 1.10 ^a^	<0.0001
3	Chlorogenic acid	136.3 ± 1.27 ^b^	327.6 ± 3.68 ^a^	<0.0001
4	Catechin	102 ± 1.32 ^b^	400.2 ± 6.13 ^a^	<0.0001
5	*p*-Hydroxybenzoic acid	138.5 ± 1.58 ^b^	587.9 ± 4.89 ^a^	<0.0001
6	Caffeic acid	13.28 ± 0.60 ^b^	77.37 ± 0.66 ^a^	<0.0001
7	Vanillic acid	26.59 ± 0.81 ^a^	23.53 ± 1.10 ^b^	0.0114
8	Syringic acid	11.25 ± 0.72 ^a^	8.57 ± 0.49 ^b^	0.001
9	Rutin	nd	nd	-
10	Ellagic acid	100.5 ± 3.62 ^b^	362.6 ± 2.80 ^a^	<0.0001
11	*p*-Coumaric acid	35.9 ± 0.53 ^b^	124.2 ± 2.73 ^a^	<0.0001
12	Vanillin	nd	nd	-
13	Ferulic acid	11.61 ± 0.32 ^b^	27.3 ± 0.53 ^a^	<0.0001
14	Rosmarinic acid	nd	nd	-
15	Salicylic acid	nd	nd	-
16	Methylparaben	47.12 ± 1.04 ^b^	271.6 ± 3.21 ^a^	<0.0001
17	Quercetin	nd	nd	-
18	Cinnamic acid	124.2 ± 0.44 ^b^	274.3 ± 1.00 ^a^	<0.0001

^1^ Each value is expressed as means ± SD of three independent measurements. * *p* < 0.05 indicates that the mean value of at least one component differs from the others. For each mushroom sample, means within a line with different letters differ significantly (*p* < 0.05). nd = not detected.

**Table 3:** Biomolecule groups of the derivatized methanolic extracts by GC–MS analysis.

Compound Names	*P. flaccida* (%)	*L. nuda* (%)
Sugar compositions	52.51	22.88
Fatty acids	11.71	29.72
Amino acids	16.03	18.29
Organic acids	10.53	11.11
Other groups	9.21	17.97
Total	99.99	99.97

**Table 4:** EC_50_ (mg/mL) of antioxidant properties of the methanolic extracts from Northern Morocco and of the standard Trolox^®^.

Assays	*P. flaccida* (mg/mL)	*L. nuda* (mg/mL)	Trolox (mg/mL)	One-Way ANOVA *
DPPH radical-scavenging activity	1.18 ± 0.11 ^a^	0.98 ± 0.01 ^b^	0.020 ± 0.01 ^c^	<0.0001
β-carotene/linoleate assay	0.22 ± 0.01 ^b^	0.39 ± 0.02 ^a^	0.006 ± 0.01 ^c^	<0.0001
Ferricyanide/Prussian blue assay	0.63 ± 0.01 ^a^	0.48 ± 0.00 ^b^	0.080 ± 0.02 ^c^	<0.0001

The results are presented as mean ± SD (n = 3). * *p* < 0.05 indicates that the mean value of at least one component differs from the others. For each mushroom sample, means within a line with different letters differ significantly (*p* < 0.05).

## Data Availability

Data is contained within the article or Supplementary Material.

## Supplementary Materials

The following supporting information can be downloaded at: https://www.mdpi.com/article/10.3390/molecules28031123/s1, Figure S1: HPLC–MS chromatogram of phenolic compounds in *Lepista nuda* extract detected at 280 nm, Figure S2: GC–MS chromatogram of *P. flaccida* derivatized methanolic extract, Table S1: Sugar compositions of the derivatized methanolic extract by GC–MS analysis, Table S2: Fatty acids of the derivatized methanolic extract by GC–MS analysis, Table S3: Amino acids of the derivatized methanolic extract by GC–MS, Table S4: Organic acids of the derivatized methanolic extract by GC–MS analysis, Table S5: Rest of the biomolecule constituents of the derivatized methanolic extract by GC–MS analysis, Figure S3: Radical-scavenging activity on DPPH radicals. Each value is expressed as mean ± SD (n = 3), Figure S4: Lipid peroxidation inhibition measured by the β-carotene bleaching inhibition. Each value is expressed as mean ± SD (n = 3), Figure S5: Reducing power. Each value is expressed as mean ± SD (n = 3).

## Sample Availability

Samples of the compounds are not available from the authors.
