# Supplementary material for: Antioxidant Properties, Bioactive Compounds Contents, and Chemical Characterization of Two Wild Edible Mushroom Species from Morocco: Paralepista flaccida (Sowerby) Vizzini and Lepista nuda (Bull.) Cooke
Source: Molecules. 2023 Jan 23;28(3):1123. doi: 10.3390/molecules28031123 (PMC9920256; doi:10.3390/molecules28031123)
Supplement: Supplementary file 1 [file molecules-28-01123-s001.zip › molecules-2158325-supplementary.pdf]

Supplementary File

Antioxidant Properties, Bioactive Compounds Contents, and Chemical Characterization of Two Wild Edible Mushroom Species from Morocco *Paralepista flaccida* (Sowerby) Vizzini and *Lepista nuda* (Bull.) Cooke

El Hadi Erbiai [1,2], Abdelfettah Maouni [1], Luís Pinto da Silva [2,3], Rabah Saidi [1], Mounir LEGSSYER [1], Zouhaire Lamrani [1] and Joaquim C. G. Esteves da Silva [2,*]

1 Biology, Environment, and Sustainable Development Laboratory, Higher School of Teachers (ENS), Abdelmalek Essaadi University, 93000 Tetouan, Morocco; elhadi.erbiai@etu.uae.ac.ma (E.H.E.); amaouni@uae.ac.ma (A.M.); r.saidi@uae.ac.ma (R.S.); mlegssyer@uae.ac.ma (M.L.); zlamrani@uae.ac.ma (Z.L.)

2 Chemistry Research Unit (CIQUP), Institute of Molecular Sciences (IMS), Department of Geosciences, Environment and Territorial Planning, Faculty of Sciences, University of Porto, Rua do Campo Alegre s/n, 4169-007 Porto, Portugal; luis.silva@fc.up.pt

* Correspondence: jcsilva@fc.up.pt; phone: +351 220402569.

SUPPLEMENTAL INFORMATION

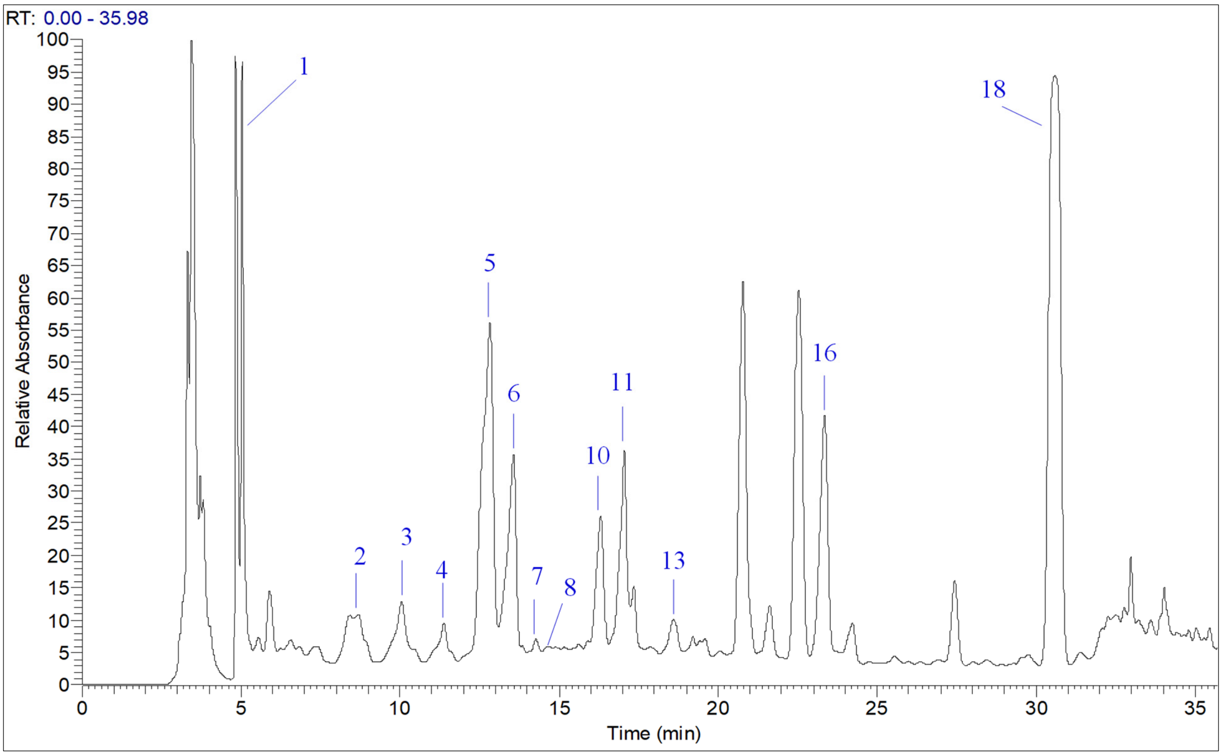

Figure S1: HPLC–MS chromatogram of phenolic compounds in *Lepista nuda* extract detected at 280 nm.

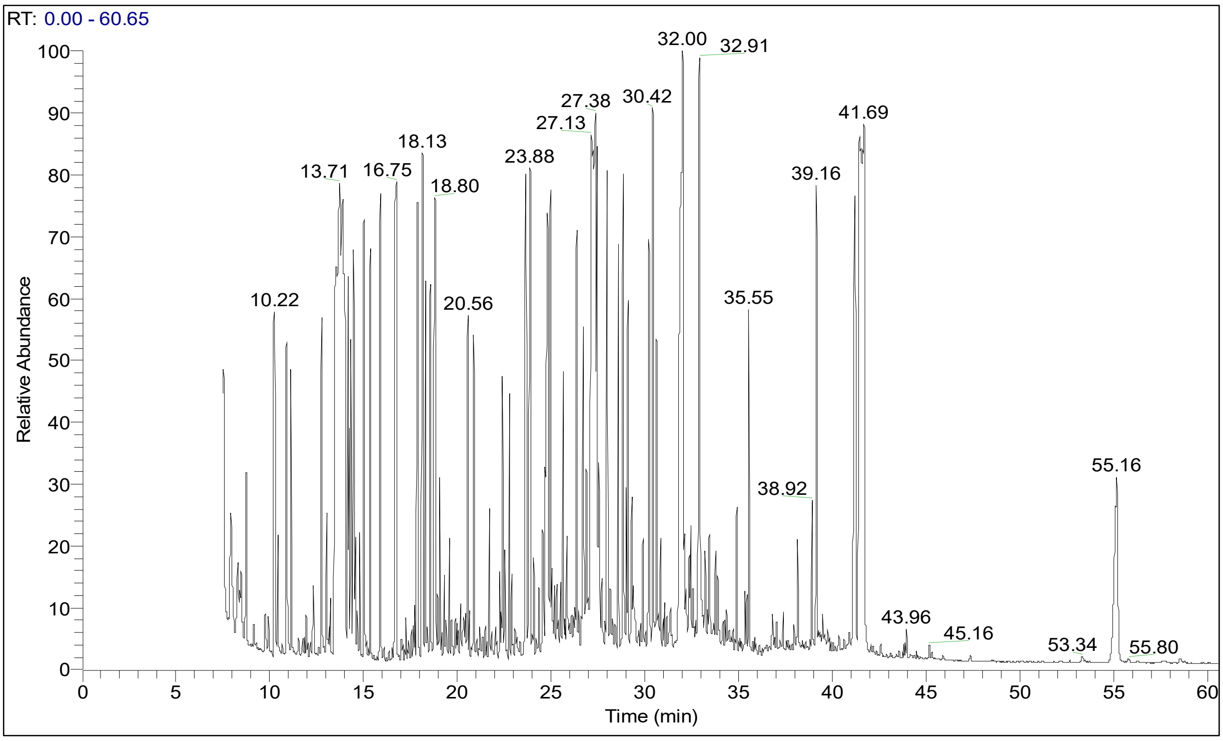

Figure S2: GC–MS chromatogram of *P. flaccida* derivatized methanolic extract.

Table S1: Sugar compositions of the derivatized methanolic extract by GC-–MS analysis.

Compound names	*P. flaccida*		*L. nuda*	
	**Area %**	**Kovats' RI***	**Area %**	**Kovats' RI***
Glycerol	**12.42**	1275	-	-
3-Deoxy-d-mannitol	-	-	1.66	1828.79
Arabinitol	0.81	1737.81	0.27	1719.40
Cellobiose	0.13	2921.99	-	-
D-Allose	2.86	1918.23	0.13	1976.04
D-Fructose	2.43	1846.97	-	-
D-Galactose	0.89	2294.08	0.99	2077.32
D-Gluconic acid	0.81	2034.02	0.48	2013.40
D-Glucose	-	-	1.41	2373.94
D-Ribonic acid	0.16	1802.53	-	-
Erythritol	0.69	1516.42	1.61	1498.98
Glyceric acid	0.18	1333.87	-	-
Inositol	1.45	2120.11	0.22	2096.91
Lactulose	1.09	2060.31	-	-
L-Arabinose	0.34	1891.41	-	-
L-Fucose	0.12	1711.44	-	-
L-Sorbose	-	-	0.11	2679.75
Mannitol	**10.39**	1971.35	**5.16**	1943.75
Mannobiose	-	-	1.78	2628.48
N-Acetyl-D-galactosamine	1.88	2131.61	-	-
N-acetyl-D-galactosaminitol	0.89	2143.10	0.18	2117.65
N-Acetyl-D-glucosamine	0.5	2101.72	0.32	2107.47
Threitol	2.69	1507.96	**4.16**	1492.86
Threonic acid	0.28	1554.23	0.27	1536.32
Trehalose	**8.58**	2814.78	**4.13**	2777.27
Turanose	2.92	2789.20	-	-

*Kovats' RI: Kovats retention index of compound's derivative form.

Table S2: Fatty acids of the derivatized methanolic extract by GC–MS analysis.

Compound names	*P. flaccida*		*L. nuda*	
	**Area %**	**Kovats' RI***	**Area %**	**Kovats' RI***
3-Methylbutanoic acid	0.16	956.33	-	-
10,12-Docosadiynedioic acid	-	-	0.11	3324.14
2-Hydroxyisocaproic acid	0.25	1235.80	-	-
5,8,11,14-Eicosatetraynoic acid	-	-	0.72	2576.97
Behenic acid	-	-	0.1	2612.03
Lauric acid	-	-	0.12	2620.25
Lignoceric acid	-	-	0.2	2808.37
Linoelaidic acid	-	-	**21.13**	2193.63
Linoleic acid	**9.67**	2223.08	-	-
Nervonic acid	-	-	0.09	2788.64
Palmitic acid	**1.63**	2047.42	**4.49**	2029.90
Palmitoleic acid	-	-	0.65	2007.22
Pentadecanoic acid	-	-	0.33	1927.60
Stearic acid	-	-	**1.78**	2209.35

*Kovats' RI: Kovats retention index of compound's derivative form.

Table S3: Amino acids of the derivatized methanolic extract by GC–MS.

Compound names	*P. flaccida*		*L. nuda*	
	**Area %**	**Kovats' RI***	**(Area %)**	**Kovats' RI***
Alanine	1.22	1109.64	**2.15**	1093.79
Asparagine	0.25	1684.80	-	-
Aspartic acid	1.01	1528.86	0.57	1510.45
gamma-Aminobutyric acid	**3.04**	1541.29	-	-
Glutamic acid	0.82	1627.94	1	1609.80
Glutamine	**1.99**	1792.04	-	-
Glycine	0.54	1123.49	0.47	1106.63
Histidine	0.66	1945.31	-	-
Isoleucine	1.06	1301.61	1.56	1283.52
Leucine	-	-	**9.05**	1257.95
Lysine	0.98	1936.46	-	-
Phenylalanine	0.78	1643.63	0.8	1624.02
Proline	1	1309.14	-	-
Threonine	**1.42**	1393.55	**2.69**	1376.88
Tryptophan	0.31	2250.30	-	-
Valine	0.95	1221.59	-	-

*Kovats' RI: Kovats retention index of compound's derivative form.

Table S4: Organic acids of the derivatized methanolic extract by GC–MS analysis.

Compound names	*P. flaccida*		*L. nuda*	
	**Area %**	**Kovats' RI***	**Area %**	**Kovats' RI***
2-Hydroxyglutaric acid	0.16	1579.60	-	-
3,4-Dihydroxybutanoic acid	**2.59**	1436.73	-	-
Acetoacetic acid	-	-	**2**	1418.37
Chinchomeronic acid	-	-	0.72	1223.30
Citric acid	**1.83**	1838.89	0.48	1816.67
Glycolic acid	0.15	1081.37	0.45	1064.60
Lactic Acid	**1.56**	1068.32	**1.32**	1052.17
Maleic acid	-	-	**1.44**	1328.49
Malic acid	**2.26**	1495.92	0.51	1475.51
Methylsuccinic acid	0.13	1327.42	**1.2**	1520.90
Oxalic acid	-	-	**1.66**	1205.11
Sebacic acid	-	-	0.64	2506.58
Succinic acid	**1.85**	1316.13	-	-
Tartaric acid	-	-	0.69	1935.42

*Kovats' RI: Kovats retention index of compound's derivative form.

Table S5: Rest of the biomolecule constituents of the derivatized methanolic extract by GC—MS analysis.

Compound names	*P. flaccida*		*L. nuda*	
	**Area %**	**Kovats' RI***	**Area %**	**Kovats' RI***
1,3,8-Trihydroxyxanthone	-	-	0.13	918.35
19-Norandrosterone	-	-	0.31	2393.94
1-Tetracosanol	-	-	0.23	2949.79
2,3-Butanediol	0.08	1049.69	1.28	1291.48
2,4,7-Trimethyl-1,8-naphthyridine	-	-	1.4	960.13
2-Monoolein	-	-	0.18	2929.88
5-Chloro-1H-indole-2,3-dione	-	-	0.3	1116.87
5-Methyluridine	0.65	2437.34	0.14	2408.23
Adenosine	**1.24**	2669.62	0.1	2638.61
Carbodiimide	0.55	975.95	-	-
Chloro-(3-chloropropyl)-dimethylsilane	0.35	948.10	**3.62**	932.28
Deanol	0.64	924.05	0.24	909.49
Dehydroabietic acid	-	-	0.15	2341.21
Diethylene glycol	0.15	1243.75	-	-
Digoxigenin	-	-	1.02	1385.48
Ergosterol	**1.97**	3267.51	**1.61**	3236.38
Ethanolamine	0.12	1038.51	**1.85**	1298.30
Glycerol monostearate	-	-	0.21	2761.93
Glycerophosphoric acid	**2.44**	1781.59	0.6	1760.70
Glyceryl palmitate	-	-	0.08	2569.74
Inosine	0.23	2606.33	-	-
Phosphoric acid	-	-	**4.14**	1271.02
Thymine	-	-	0.38	1864.65
Trifluoroacetamide	0.63	898.71	-	-
1,1,1-Trifluoro-3,3-bis(trimethylsilyl)propan-2-one	0.16	959.49	-	-

*Kovats' RI: Kovats retention index of compound's derivative form.

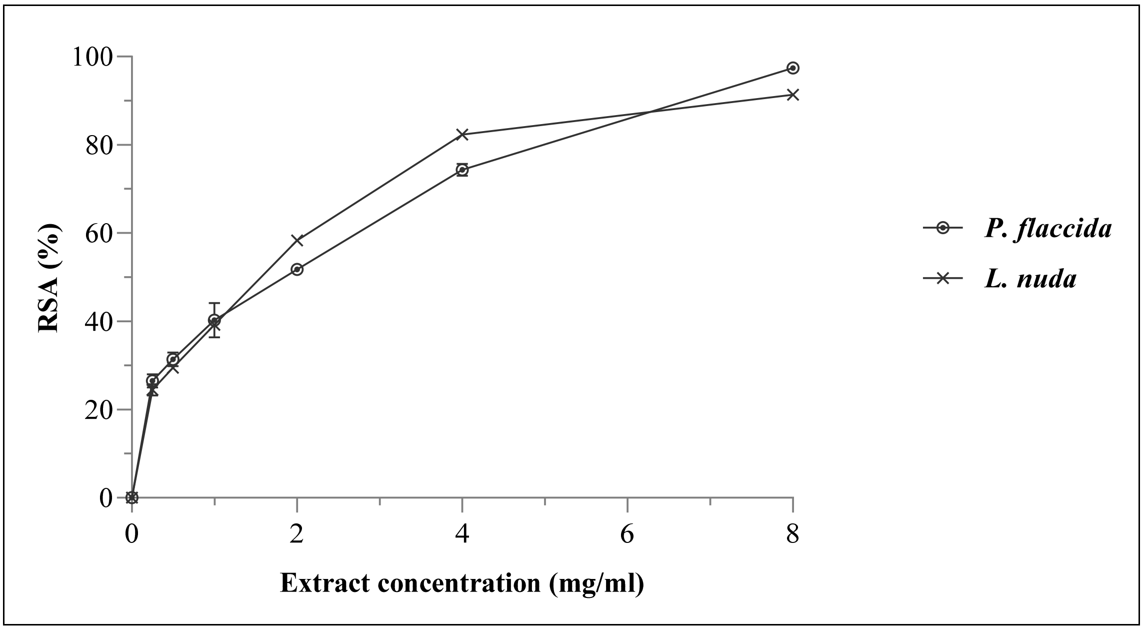

Figure S3: Radical-scavenging activity on DPPH radicals. Each value is expressed as mean ± SD (n = 3).

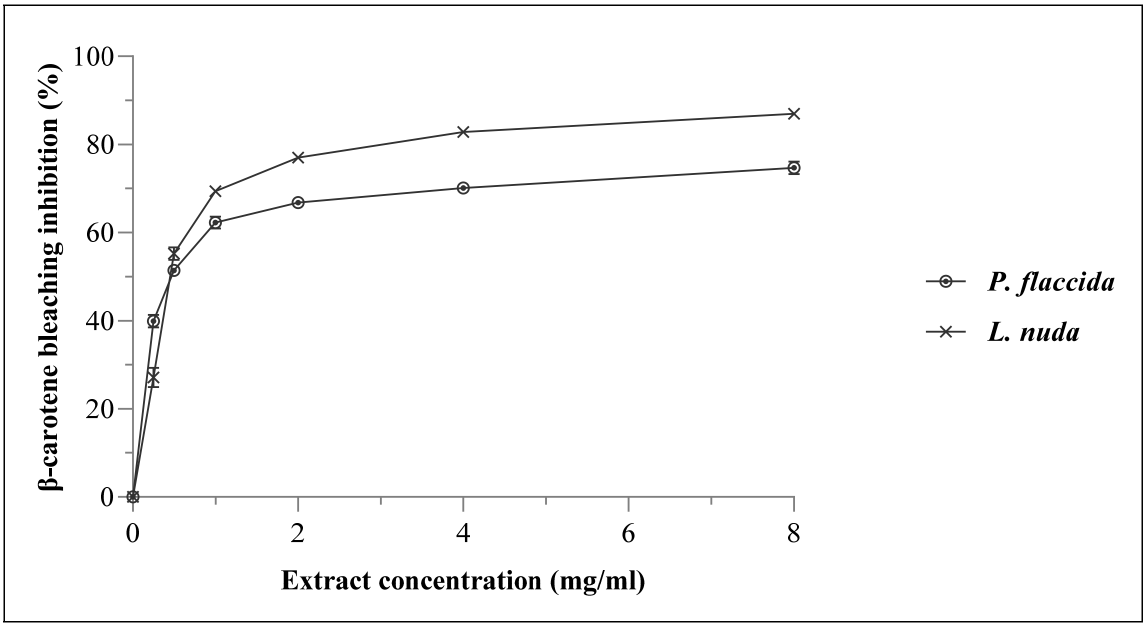

Figure S4: Lipid peroxidation inhibition measured by the β-carotene bleaching inhibition. Each value is expressed as mean ± SD (n = 3).

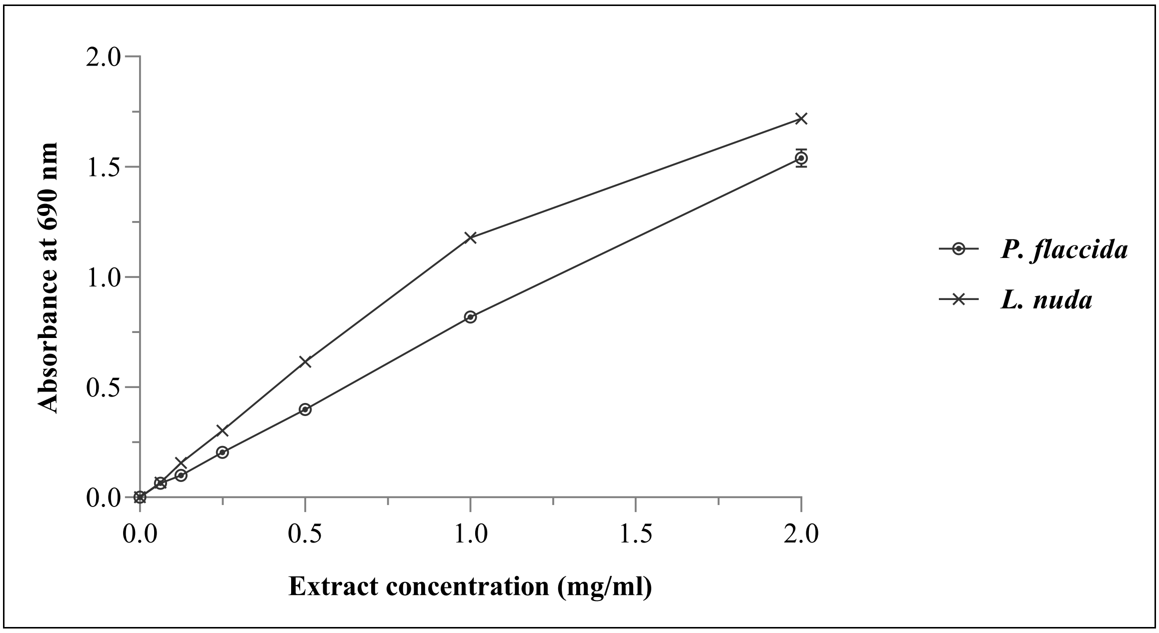

Figure S5: Reducing power. Each value is expressed as mean ± SD (n = 3).
